# The Treatment of Mental Cases by the General Practitioner

**Published:** 1910-01-15

**Authors:** Robert Jones

**Affiliations:** Lecturer on Mental Diseases, St. Bartholomew's Hospital, London


### Hospital Clinics.

### THE TREATMENT OF MENTAL CASES BY THE GENERAL PRACTITIONER.

By ROBERT JONES, M.D., F.R.C.P.; Lecturer on Mental Diseases, St. Bartholomew's Hospital, London.

(A Lecture delivered at the Post-Graduates' College, West London Hospital, on December 7, 1909.)

I wish to direct my remarks particularly to the treatment of insanity before it reaches the asylum—that is, insanity in the hands of the general practitioner. A good many practitioners have anticipated the law by taking into their houses cases of incipient insanity. The law at present does not allow that, but medical men think they are acting humanely in not sending the patients to an asylum; and certainly, if attacked by insanity, I should greatly prefer to get well quickly under the care of a general practitioner in his private house rather than have resting upon me the stigma of having been an inmate of an institution for the insane.

### Residences.

The private house—the medical man's house—generally comes under the legal term "institution." There are four kinds of institutions recognised by law. (1.) The asylum proper, being the county or borough asylum, or the district asylum in Scotland or Ireland. (2.) The registered asylum, such as Bethlem and St. Luke's. Altogether there are fourteen of these institutions in England and Wales. The registered hospitals were originally, no doubt, places of private philanthropy, and now the surplus of income over expenditure supports the poor insane. (3.) The licensed house, which is really a private asylum. The private asylum can only receive a limited number of patients, but powers have been given to local authorities, such as the London County Council, to build such accommodation for private patients if they feel so disposed. (4.) Unlicensed houses for single care, the one patient they are allowed to take being duly certified. But, in spite of this, there are a great number of uncertified patients in the incipient stage of insanity who are cared for in private houses, and when there is an amendment to the Lunacy Act, as there is bound to be before long, I think it will permit treatment of incipient lunacy in private houses with the full knowledge and consent of the Commissioners in Lunacy.

### Certificates.

The doctor's certificate in cases of insanity is an important document. It confers upon the medical man exceptional powers. He can deprive a man of his civil, social, domestic, political, and financial liberty. And therefore it is exceedingly necessary that, although the medical man is not expected to know every form of insanity, he should know enough not to take away a man's liberty unnecessarily. A practical point of importance to the medical man which I should like to impress upon him is that in the medical certificate he must not use queer terms, such as irritable, emotional, bad tempered. Great care must be taken as to every detail. I have had certificates sent back because they did not state the number of the house, or because the "9" was left out of "1909." It is really most essential that the certificate should be filled in carefully, and the symptoms should be stated so obviously plainly and clearly that they will convince the most obtuse layman. A certificate holds good for seven days—that is to say, if it is presented within seven days of signature it is all right. In my own experience I have found cases of typhoid fever, of pleurisy and pneumonia brought forward as cases of insanity. One girl in the early stages of delirium was brought to me as insane; a day later she developed the rash of scarlet fever. I have had cases sent to me as cases of insanity which were really acute meningitis, and this condition, with the vomiting, the contraction of the pupil and the temperature, should not be difficult to distinguish in diagnosis. Scores of people who have been suffering from the delirium due to alcohol have been sent to an asylum. It is, of course, a very severe lesson to a man to wake up out of a drunken delirium and find himself in such an institution. Indeed, it is hard lines upon him, for his social recovery is thereby rendered more difficult. He comes out of the asylum with a stigma upon him. He cannot get back to his previous employment. He is unable to get a living on the same plane as before, and therefore he descends to a lower one. I have seen more than one such case.

### Diagnosis.

It is important, therefore, to diagnose between delirium and insanity. The first thing is to find out when the delirium takes place. If at night, when the patient is in a drowsy state, with high temperature, furred tongue, rapid pulse, and highly-coloured urine, we may know that these are not usually the accompaniments of insanity. In dealing with insanity, too, an important factor is the pupil test. Then I think you ought also to stay your hand with puerperal cases—cases of puerperal insanity occurring within six weeks of delivery. Every effort should be made to treat these privately at home; otherwise, if the woman becomes pregnant again, she may recall the former circumstances, and we shall have her under care earlier and for the insanity of pregnancy. Therefore, in toxæmic cases, such as are puerperal ones, as well as with "young" people suffering from "acute mental trouble from some one great cause," and in cases where there is a history of traumatism, especial care should be taken to withhold, if possible, the stigma of the asylum.

### Examination.

In examining a patient it is really important, I think, that a medical man should not have occasion to disguise his identity. I have gone with the general practitioner in my neighbourhood—the East End of London—to examine various people, and I have never been introduced as the "clock-winder"! I have never found any difficulty in introducing myself as a doctor interested in a patient's mental condition. Some hints can be gathered from an examination of his room. He may have barricaded the doors and locked the windows. Then there is a peculiarity about his physiognomy. He has a vacant stare, or an anxious look. There may also be something strange about his dress. The lunatic of a certain type decorates himself, believing that he is a very important personality, or he wears some little ribbon "to save my life." The first thing I say is, "Do you answer them back?" "What do you mean?" he will ask; but by degrees you will generally find that he will admit to aural hallucinations. If you sympathise with him he will probably tell you all about his delusions of persecution. If, however, he will not talk, ask him to write to you. The letter will often convey more than the ordinary interview. A great many people in asylums are rather keen upon seeing the doctor, and yet when they see him they will not speak, but will hand him a letter. Only this morning I had a small letter of twenty-three pages handed to me for inspection by a person who refuses to speak to me.

### Symptoms.

It is very important to watch for suicidal tendencies. Recently a woman came from Somersetshire to see me, accompanied by a sister. She declared that she had nothing the matter with her, but that her "instincts" were changed. She felt a loss of confidence in herself—the *folie du doute*. Persons in this condition cannot form conclusions and inferences from premises. She had a general numbness of sensibility. By testing the different nerves neurologically I could make out really nothing. The patient herself suggested at the end of the interview that the *x*-rays should be brought to bear upon her brain. This, however, could hardly be satisfactory in such a case, although I believe the rays are used, and even percussion is employed, to find out the position of cerebral tumours. The family history is frequently of assistance. This woman was the daughter of an epileptic, and epilepsy tends to be inherited, not as epilepsy, but as one of the various neuroses that may interchange with insanity. Epilepsy very often enters into the ancestry of people who are whimsical and peculiar or who suffer from hysteria.

### Home Treatment.

It is a good rule to have home treatment as long as possible. The home treatment of insanity, however, is very expensive. It is necessary to have at least two nurses; and, in fact, insanity is the most expensive illness with which anyone can be afflicted! If you have single care—and I am applied to not infrequently by medical men and others for quiet patients, and I have sent a fair number of these patients out at low rates, even at £100 per year, as single cases—you must have two medical certificates, a petition, and an order.

### Legal Forms.

There must be a Justice's order for all private patients, as required by the Lunacy Act of 1890. The urgency order and the medical certificate hold good for seven days. And whether it is a case for single care or a case for an institution, it is necessary to have either this urgency order and medical certificate or the two medical certificates, the petition, and the order. The urgency order must be made by a relative who has seen the patient within two days. The certificate is signed by the medical attendant, who also must have seen the patient within two days, and the patient must be admitted with the order within two days of its being signed. The order is in force for seven days, and then it is necessary to have the petition, the order, and the two medical certificates. If, however, these are said to be "pending," the urgency certificate may be more or less indefinite in its period of enforcement.

### Psychology.

It is my firm opinion that the medical man ought to know something of psychology. A little book from the pen of Miss Brackenbury is sold at ninepence—I have no interest in its publication—which is called "A Primer of Psychology," and forms an excellent guide to the subject and a useful introduction to William James and others. It is only by the knowledge of psychology that the medical man is able to make mental analyses and see how mental processes combine, and how afterwards they can be resolved. In America it is not uncommon for commercial men, medical men, novelists, and teachers to attend psychology classes. Many years ago I was one of Sully's pupils at University College, and although he was the highest authority on the subject, only four of us formed his class during that session, and I think the sexes were evenly divided amongst us. But in America and Germany psychology is taught fairly extensively, and is studied even by such people as poets and novelists who want to learn the whole gamut of emotions and passions. Some knowledge of psychology is essential to men in asylum practice, and I believe that before very long we shall have a diploma for medical men in psychological medicine, just as we now have the D.P.H. for health officers, and that in the future no medical man will be appointed to an asylum unless he has that diploma.

### Law Courts.

If a medical man enters a court of law, the first thing he is asked to do may be to define insanity. He is unwise to attempt a definition. I have been heckled often to define insanity, but it is much easier to describe it than to define it. There are certain conditions and circumstances which it is necessary to consider before one can appreciate insanity, and certainly before any sort of definition is possible. For instance, the *joie de vivre*, which in young people is quite natural, is a suspicious circumstance when it makes its appearance in elderly or middle-aged persons. Financial circumstances and social conditions have to be taken into account. The insanity of uneducated people has a different manifestation from that of the educated. The surroundings and the time at which the symptoms occur have to be considered. There are only two avenues along which we arrive at a full knowledge of one another—namely, conduct and conversation.

A very good idea of the analysis of conduct is to be obtained from Herbert Spencer's "Sociology." Conduct he describes as being directly self-preservative and also indirectly self-preservative. It is directly self-preservative in so far as it relates to certain instincts planted within us—the seeking for food, the desire for warmth, and the gratification of the sex instinct, for example. It is indirectly self-preservative in its relation to certain social performances—to the regard we pay to certain duties as people who live in a community; for instance, the payment of rates and taxes and service on a jury. Society establishes certain rules and regulations which ordinary men acquiesce in. A failure to comply with these on the part of one member of society necessitates punishment. If he wishes to escape punishment he puts forward some exculpatory plea.

### History.

This leads up to the question of responsibility, and responsibility is very delicate ground. At this point comes in the law. It is necessary to place the man and his property under control, and in doing that we must invoke legal aid. Able men have tried to define insanity legally. Hale started the definition that if a grown-up person had the faculties of a child of fourteen he was insane. Two things were compared in that definition—diseased maturity and healthy immaturity—which were not properly comparable. Another legal luminary suggested degradation of the understanding and memory to such an extent that a man might become equal to a brute or a beast or be an infant. Mansfield came on the scene and laid down a dictum about the knowledge of right and wrong, and that has also been a long and vexed question. Tindal suggested that there was such a thing as partial insanity—that the will could be diseased and not the reasoning power. This, in reality, was the first introduction to moral insanity, and the Royal Commission on Feeble-mindedness publicly discovered moral imbecility, although, of course, it had been recognised by experts long before that Commission. But the Commission gave it a place in the public view, and the result ultimately will be without doubt that a special colony or home will be provided for these moral imbeciles. Mansfield, therefore, was actually the first to draw attention to moral insanity. Fitz-Stephen, reasoning upon the same subject, asked if a man could know the nature of an act when he could not pass a rational judgment upon its moral character. Later still, Section 116 of the present Lunacy Act recognises that insanity may be the accompaniment of disease and age. This is the great practising section for lawyers.

The first knowledge that we possess of the care of the insane belongs to the time of Edward II. At that time the custody of the insane belonged of right to the Crown. If the person was an idiot the Crown took away all his property subject to providing him with necessaries, and if he was a lunatic the Crown had supervision of his property and the King acted as his trustee. The first of these conditions lapsed because the people did not like to see the Crown take away the property of private individuals. The second still remains in force, the Lord Chancellor acting as the trustee for every lunatic.

### Legal Forms of Insanity.

Lawyers used to distinguish *fatuitas naturalis* from *non compos mentis* by finding out whether the patient could count twenty, knew his age and his father and mother. For a long time this definition of feeble-mindedness remained. The term *non compos mentis* is scarcely ever applied to idiots now.

Since the Idiots Act of 1886 we no longer look upon an idiot as *non compos mentis*. The Act recognises that idiots are educable. Therefore Coke's idea of perpetual infirmity has been cast on one side. Lunacy proper can generally be divided under three terms—namely, illusions, delusions, and hallucinations. An illusion is a false interpretation. There is always an object for an illusion, but there is none for a hallucination. The hallucination is really a deeper mental reduction than the illusion. A delusion is a false belief. It has nothing to do with the senses. There are many people who have pure delusions. It is the ordinary popular conception of madness. A man who has one delusion is called a monomaniac. These perversions of the senses and of the impressions form a sub-stratum in the minds of the insane, and this, in its turn, forms motives. The relation of motives to actions is the same as in the sane. Dr. Johnson, Julius Cæsar, and John Gilpin, as well as many others, are supposed to have had delusions.

### Varieties.

What are we to do with a case of insanity? As long as it is possible it should be treated at home. Let us consider for a moment the different groups of lunatics. There is first of all the criminal lunatic, who is already legally in chains. There are two classes of criminal lunatics: (1) persons who become insane during their sentence, and who are called the Home Secretary's lunatics, and (2) those who are found insane on arraignment, and are called the King's lunatics because they are detained during His Majesty's pleasure. These two classes do not concern us at all. Then there is the pauper class. Any lunatic is a pauper who cannot afford to pay for private accommodation. Usually the minimum is about £1 per week, so that the person who does not possess this income may become a pauper lunatic. There are four kinds of paupers. (1.) People not under proper care and control—*i.e.* who are cruelly treated and neglected. (2.) Resident pauper lunatics. (3.) People wandering at large—a not inconsiderable class. They are placed in an asylum until their identity is disclosed. The appearance of the wanderer must not be taken to indicate his actual position. I know the case of a man who lived in the southern counties and had delusions of persecution. He came up to London in his hunting-suit, having first put £50 in notes in each of his top-boots. Arrived in London, he changed his clothes with a vagrant beggar and wandered away. He was found in a deplorable condition, and was brought to me as a pauper lunatic wandering at large. It was some time afterwards before his identity was disclosed; then, of course, being a well-to-do man, he was no longer a pauper lunatic. No petition is required in this class of cases. Somebody gives information to the police, or the relieving officer, and the man is apprehended and taken before a justice of the peace, who calls in a doctor to certify to his condition, and he is taken to the asylum. (4.) The fourth class of paupers comprises those who are in workhouses.

### "Boarders."

There is also a section of people who may be called "voluntary boarders." Anyone who feels an attack of insanity coming on, or who fears impending mental breakdown, can go voluntarily into a private asylum or hospital, such as that of Bethlem or St. Luke's, be detained there, and leave whenever he pleases by giving twenty-four hours' notice. But—and here the trapping part comes in —if the Commissioners care to have him examined he can be certified and held as an insane person irrespective of any notice or action on his part to get away.

### Travel.

Just one word about travelling. Many neurologists send their patients on a sea-voyage. A friend of mine who was going round the world with his little boy wrote to me from Ceylon that between home and Colombo two valuable lives had been lost. They were men who had been sent travelling by the neurologist and had precipitated themselves overboard. The question of travel for a particular patient requires more thought and care than is usually given to it.

### Restraint.

Before I come to the question of treatment, I may add that you cannot use mechanical restraint for a certified patient except for medical and surgical reasons, or to avoid danger to himself and others, and when restraint is employed for these purposes, the matter must be reported to the Commissioners. There is not a "strait-waistcoat" in use at any asylum. If one is there at all, it is merely shown as a curiosity. I have never seen one at an asylum. Moreover, the patients are turned out into the gardens and allowed to roam about, which they do very freely when they find that no one is taking notice of them. Indeed, the restraint in an asylum is not the overwhelming thing that many outsiders believe.

### Treatment.

Now we come to the question of treatment. It was Shakespeare who suggested the development of the insane mind. The passage occurs in "Hamlet," Act II., Scene 2:—

> "And he, repulsèd (a short tale to make),
> Fell into a sadness; then into a fast;
> Thence to a watch; thence into a weakness;
> Thence to a lightness; and, by this declension,
> Into the madness wherein now he raves,
> And all we wail for."

Here we have the successive stages—melancholia, the refusal of food, sleeplessness, and delirium—until we reach mania. It is an excellent description of incipient insanity. A great deal is scoffingly talked about "asylum routine," but I believe asylum routine is an excellent method of waking up rational thought and memory. There is a stigma attaching to it, but nevertheless the treatment in an asylum is the best possible treatment for confirmed mental cases. In an asylum we have nurses who have been specially trained, and who are young, watchful, alert and careful. These nurses themselves help to do the housework; and even lady private patients, when they see the nurses thus engaged, often show the first sign of improvement by beginning to take a little interest in domestic work. It is in regard to these little matters that the first signs of convalescence are noted. Very often in the case of a female patient the first sign of convalescence is in the extra care she bestows upon her hair. People in charge of the insane, either in asylums or private houses, have to follow the patients about, wait for them in the lavatories, and generally perform duties in attendance which would ordinarily be repulsive. If the patient is in a home, the medical man must keep notes as to his temperature, food, excretions, sputum, sleep, exercise, or any fresh delusions.

### Sleep.

Sleep and exercise are two very important things in the treatment of the insane, and at Claybury I keep a sleeping chart of all the patients who come into residence. There are methods of curing sleeplessness indirectly. Digitalis is very valuable in some cases. In toxæmic and puerperal cases of insanity I have freely administered beef tea, milk, and eggs, and occasionally alcohol in the form of dry wines. It is useful indirectly to affect sleep to give a little beef tea in the early hours of the morning in cases of melancholia. These cases are at their worst in the early morning, while neurasthenic cases, on the other hand, are worse at night. Chloral and bromide of potassium are the best sedatives for cases of overwork and mental exhaustion. I always advise the use of a hot-water bottle when chloral is given to the old people. Opium is a sedative-stimulant, especially good for old people. Sulphonal may also be given—20 grains in warm milk or beef tea. The worst of sulphonal is that it sets up such a great amount of intestinal irritation. I have seen the mucous membrane of the small intestine covered with what appeared to be spicules of glass after sulphonal had been given. Paraldehyde is a very valuable drug, and I use it, as well as chloral and bromide together, for certain toxæmic cases. I have used bromide of potassium in large doses when necessary, and have found the patients to whom it has been administered sleep more or less for two or three days after a series of epileptic convulsions. Hyosine hydro bromate gr. $\frac{1}{120}$ is the best sedative for violent excitement.

Motor.

Dr. C. A. Mercier tells me that he has averted insanity by sending his patient out on a long motor drive.

Forced Feeding.

Then there is the question of forcible feeding. There is such intense exhaustion through insanity that forcible feeding is absolutely necessary. Seventy per cent. of the cases of acute delirious mania that come into the asylums end fatally. Unless we feed the patient from the very moment that he comes into the asylum we will certainly lose him. It is important to have the bowels open. I have used 10 grains of calomel once, twice, even three times in some cases. The insane will not usually take tabloids; they imagine that they are being poisoned if these are offered.

Wills.

A final legal point concerns the making of wills by people who are insane outside or in an asylum. The points upon which a medical man should assure himself in such a case include the patient's ability to enumerate his property and those who have claims upon his bounty; whether, having enumerated these, he can enumerate them again several days later; whether there is any person around the patient who has undue influence over him, and also whether he has any delusions or morbid suspicions directed against the people who have a claim upon him.

